# Draft Genome Sequence of *Burkholderia andropogonis* Type Strain ICMP2807, Isolated from *Sorghum bicolor*

**DOI:** 10.1128/genomeA.00455-15

**Published:** 2015-05-14

**Authors:** Lucilene Lopes-Santos, Daniel Bedo Assumpção Castro, Laura Maria Mariscal Ottoboni, Duckchul Park, Bevan Simon Weir, Suzete Aparecida Lanza Destéfano

**Affiliations:** a Laboratório de Bacteriologia Vegetal, Instituto Biológico, Campinas, Brazil; b Centro de Biologia Molecular e Engenharia Genética, Universidade Estadual de Campinas, Campinas, Brazil; c Landcare Research, Auckland, New Zealand

## Abstract

Here, we report the draft genome sequence of *Burkholderia andropogonis* ICMP2807, a phytopathogenic bacterium isolated from *Sorghum bicolor* plants in the United States.

## GENOME ANNOUNCEMENT

*Burkholderia andropogonis* was described as a causal agent of stripe disease in sorghum (*Sorghum bicolor*) by Smith in 1911 (1) and, subsequently, was classified as *Pseudomonas andropogonis* by Stevens in 1925 (2). Based on DNA-rRNA hybridizations, it was reclassified to the genus *Burkholderia* (3). This bacterial species has an extensive geographical distribution and can cause leaf spots, streaks, or stripes in a wide range of hosts (4–9). The *B. andropogonis* strains are highly similar in their morphological and physiological properties (10) and have unique features that are absent in most plant pathogenic bacteria, such as a single polar sheathed flagellum (11) and rhizobitoxine production (12–14).

Genomic DNA was isolated from culture of *B. andropogonis* ICMP2807 (IBSBF199; LGM 2129; ATCC 23061) using the DNeasy blood and tissue kit (Qiagen, USA) on the automated QIAcube instrument (Qiagen, USA) and sequenced with a 454 GS Junior system (Roche Diagnostics, USA) at Landcare Research (Auckland, New Zealand). The sequencing generated 162,643 reads with an average read length of 464 bp, totaling 75.4 Mbp. The reads were trimmed and assembled with Newbler version 2.9 (15). The draft genome comprises 6.29 Mbp in 272 contigs with a GC content of 58.92%, a mean contig length of 22,808 bp, and an *N*_50_ of 155,724 bp.

Functional annotation in SEED subsystems was performed on the RAST server (16, 17). The annotation presented 6,047 protein-coding genes (CDSs), 48 tRNAs, and 4 rRNAs (one 5S, one 23S, and two 16S). Genome analysis revealed 9 genes for the peptide antibiotic colicin V and 94 genes associated with resistance to antibiotic and toxic compounds. Several genes of the type VII, VI, IV, II, and III secretion systems were identified. The type III secretion system is a common infection mechanism found in phytopathogenic bacteria. In addition, the presence of the *catABCD* operon, the *pca* cluster, and other genes related to the degradation of aromatic compounds indicates a possible environmental relevance of this strain. Further analyses are in progress and will be published separately.

### Nucleotide sequence accession numbers. 

This whole-genome shotgun project has been deposited at DDBJ/EMBL/GenBank under the accession number LAQU00000000. The version described in this paper is version LAQU01000000.
